# A Survey of Enhanced Cold Tolerance and Low-Temperature-Induced Anthocyanin Accumulation in a Novel *Zoysia japonica* Biotype

**DOI:** 10.3390/plants11030429

**Published:** 2022-02-04

**Authors:** Hai-Xiang Jin, Ming Jiang, Jian-Feng Yang, Zhi-Hao Wu, Long-Long Ma, Cong-Cong Wang, Chen Liang, Xin-Yi Ning, Liang-Fa Ge, Shu Chen

**Affiliations:** 1 Department of Grassland Science, College of Forestry and Landscape Architecture, South China Agricultural University, Guangzhou 510642, China; jinhaixianggzlc@hotmail.com (H.-X.J.); mingjiang@stu.scau.edu.cn (M.J.); zhhaowu@stu.scau.edu.cn (Z.-H.W.); malonglong@stu.scau.edu.cn (L.-L.M.); congcongwang@stu.scau.edu.cn (C.-C.W.); 201713010112@stu.scau.edu.cn (C.L.); nxyiii@stu.njau.edu.cn (X.-Y.N.); 2 Guangzhou Landscaping Company, Guangzhou 510440, China; yangyjf@139.com; 3 Guangdong Engineering Research Center for Grassland Science, Guangzhou 510642, China; 4 Department of Ornamental Horticulture, College of Horticulture, Nanjing Agriculture University, Nanjing 210095, China

**Keywords:** turfgrass, abiotic stress, anthocyanin biosynthesis, anthocyanin transport, RNA-seq

## Abstract

*Zoysia japonica* is a warm-season turfgrass that is extensively used in landscaping, sports fields, and golf courses worldwide. Uncovering the low-temperature response mechanism of *Z. japonica* can help to accelerate the development of new cold-tolerant cultivars, which could be used to prolong the ornamental and usage duration of turf. A novel *Z. japonica* biotype, YueNong-9 (YN-9), was collected from northeastern China for this study. Phenotypic measurements, cold-tolerance investigation, and whole-transcriptome surveys were performed on YN-9 and LanYin-3 (LY-3), the most popular *Z. japonica* cultivar in Southern China. The results indicated the following: YN-9 has longer second and third leaves than LY-3; when exposed to the natural low temperature during winter in Guangzhou, YN-9 accumulated 4.74 times more anthocyanin than LY-3; after cold acclimation and freezing treatment, 83.25 ± 9.55% of YN-9 survived while all LY-3 leaves died, and the dark green color index (DGCI) value of YN-9 was 1.78 times that of LY-3; in YN-9, there was a unique up-regulation of *Phenylalanine ammonia-lyase* (*PAL*), *Homeobox-leucine Zipper IV* (*HD-ZIP*), and *ATP-Binding Cassette transporter B8* (*ABCB8*) expressions, as well as a unique down-regulation of *zinc-regulated transporters and iron-regulated transporter-like proteins* (*ZIPs*) expression, which may promote anthocyanin biosynthesis, transport, and accumulation. In conclusion, YN-9 exhibited enhanced cold tolerance and is thus an excellent candidate for breeding cold-tolerant *Z. japonica* variety, and its unique low-temperature-induced anthocyanin accumulation and gene responses provide ideas and candidate genes for the study of low-temperature tolerance mechanisms and genetic engineering breeding.

## 1. Introduction

Zoysiagrasses are species of the genus *Zoysia* Willd. that are native throughout the western Pacific Rim and the Indian Ocean [1,2]. Although this genus contains 11 species, only 3 of them, *Z. japonica* Steud., *Z. matrella* (L.) Merr., and *Z. pacifica* (Goudswaard) Hotta and Kuroki, previously known as *Z. tenuifolia*, and their interspecific hybrids, have been widely used for various turfgrass applications, including home lawns, golf course tees, fairways and greens, road sides and medians, and other recreational sites [3,4,5]. These zoysiagrass varieties have acquired favor among turfgrass practitioners due to their minimal cultural demands, water-use efficiency, and extensive tolerance to cold, shade, salt, diseases, and insects [5,6]. 

Research progress in zoysiagrass have been relatively slow when compared to major agricultural crops, mostly because *Zoysia* is a perennial, open-pollinated, polyploid grass, making it genetically complicated and a challenging candidate to study. Other factors, such as insufficient funding for genomic research, limited prior omic information, major traits controlled by a small number of genes (QTL, quantitative trait loci), and a lack of information on reproducible phenotyping systems, have added extra obstacles and slowed advances in zoysiagrass research [6].

Whole transcriptome analysis based on next-generation sequencing technologies has developed as a useful approach for finding and identifying genes involved in abiotic stress resistance in recent years, drastically enhancing our understanding of the interaction between plant and environment. To date, the NCBI database holds 11 transcriptomic BioProjects for *Zoysia* species, including 8 for *Z. japonica*, 2 for *Z. matrella* [7,8], and 1 for *Z. macrostachya* [9]. Among the eight transcriptomic studies on *Z. japonica*, two focused on spike pigmentation [10], one on responses to pathogen invasion [11], and the others on responses to abiotic stresses, including aluminum, wounding [12], salt [13,14] and low-temperature [15]. 

Low temperature is a serious abiotic stress that produces reactive oxygen species (ROS), causing oxidative damage to cellular activities and endangering plant development and agricultural production. To adapt to low-temperature stress, plants have evolved intricate defensive mechanisms at the molecular, physiological, and biochemical levels [16,17,18,19], including the activation of the cellular antioxidant machinery for the purpose of scavenging ROS. Anthocyanins may scavenge ROS as an efficient antioxidant by neutralizing radicals with their hydroxyl groups to preserve normal cellular redox homeostasis; hence, plants often generate anthocyanins to reduce oxidative damage caused by low-temperature stress [20,21,22,23,24,25]. Low-temperature responsive networks in zoysiagrass have been investigated through physiological, biochemical, molecular, genetic and omic approaches [7,15,26,27,28,29,30,31,32,33,34,35,36,37]. However, no studies have attempted to associate anthocyanin accumulation with cold tolerance in zoysiagrass.

In this study, we reported a *Z. japonica* biotype collected from Lvshun West Station in northeast China and named as YueNong No. 9 (YN-9) (Figure S1), and compared its cold responses and tolerance against the most extensively used *Z. japonica* cultivar in south China, LanYin No. 3 (LY-3). The results indicated that YN-9 had better cold tolerance, accumulated anthocyanin more efficiently, and displayed unique variations of gene expressions favoring anthocyanin biosynthesis, transport, and accumulation at a lower temperature than LY-3. This study provided a novel zoysiagrass biotype with great potential for development into a cold-tolerant variety that could be used for various turf applications to extend the green period during autumn and winter, as well as candidate genes for genetic engineering breeding of zoysiagrass with enhanced cold tolerance and anthocyanin accumulation, which should advance genetic and molecular research on turfgrass and ameliorate the existing condition of a shortage of turfgrass cultivars in China.

## 2. Results

### 2.1. YN-9 Is a Zoysia japonica Biotype

**Figure 1:**
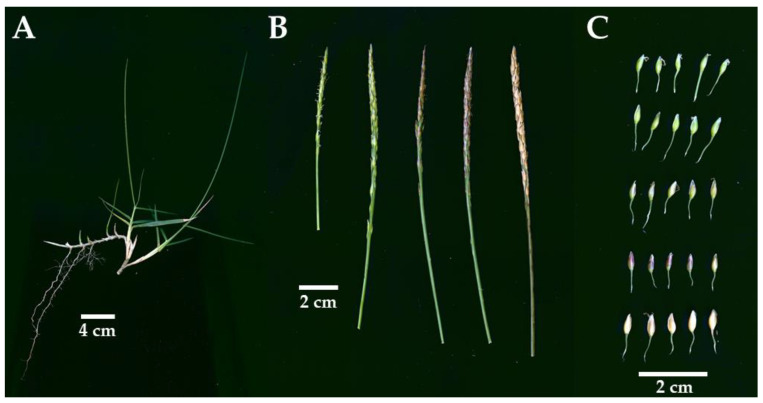
Phenotypic characteristics of YN-9 *Zoysia japonica*: (**A**) shoot and rhizome; (**B**) inflorescence and seedhead; (**C**) spikelete with pedicel.

YN-9 is a perennial grass growing with both rhizomes and stolons with the following phenotypic characteristics: the second leaves 12.47 ± 2.28 cm long and 3.92 ± 0.41 mm wide; the third leaves 14.75 ± 2.59 cm long and 3.93 ± 0.48 mm wide; inflorescences 38.20 ± 3.77 mm long and 1.92 ± 0.17 mm wide; spikelets 3.39 ± 0.47 mm long and 1.24 ± 0.16 mm wide; and pedicels 4.25 ± 1.36 mm long and usually longer than the spikelete attached (Figure 1). According to previous taxonomic publications [1,2,5], YN-9 was identified as *Zoysia japonica*. The leaf blade is the organ that most directly affects turfgrass’s aesthetic and athletic performance. Leaf width determines the texture of the turf, while leaf length determines the mowing height. Thus, the leaf length and width of YN-9 were compared to those of LY-3, the most extensively utilized *Z. japonica* cultivar for urban greening and sports fields in Southern China. The results indicated that the second and third leaf blades of YN-9 were 22.37% and 23.63% longer than that of LY-3, respectively, but no significant differences in leaf widths were observed (Table 1).

### 2.2. Enhanced Anthocyanin Accumulation and Cold Tolerance in YN-9

**Figure 2:**
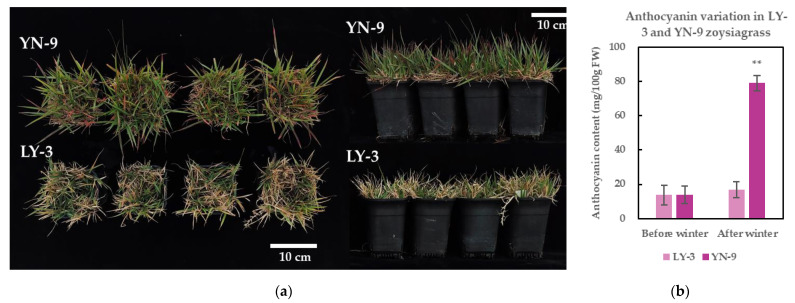
Leaf color and anthocyanin content variations in LY-3 and YN-9 after winter in Guangzhou. (**a**) Photos of LY-3 and YN-9 after winter in Guangzhou; (**b**) Anthocyanin content of LY-3 and YN-9 leaves before and after winter in Guangzhou; ** above the error bar indicates a significant difference (*p*-value < 0.01) between LY-3 and YN-9.

When LY-3 and YN-9 were grown in the natural low temperature of winter in Guangzhou (Table S1), YN-9 produced significantly more red to reddish–purple leaf blades than LY-3 (Figure 2a). Anthocyanin is measured because it is the primary source of red and purple pigmentation in plant tissues. The results showed that the anthocyanin content of YN-9 leaves after winter increased to 5.74 times that of before winter, and was significantly higher than that of LY-3 (Figure 2b), which is consistent with their leaf color variation after winter.

**Figure 3:**
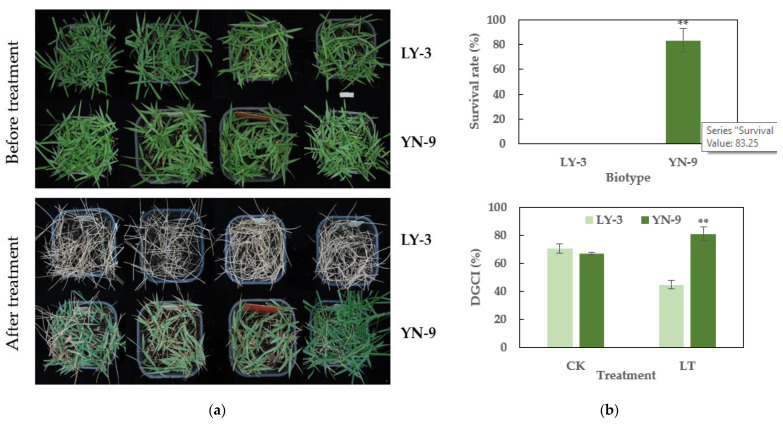
Phenotypic variations and survival rates of LY-3 and YN-009 after low-temperature treatment. (**a**) Photos of LY-3 and YN-9 before and after low-temperature treatment; (**b**) Survival rates and DGCI variations of LY-3 and YN-9 after low-temperature treatment; DGCI represents for dark green color index, ** indicates a significant difference (*p*-value < 0.01) between LY-3 and YN-9, CK indicates DGCI values before treatment, LT indicates DGCI values after treatment.

YN-9 was collected in Lvshun West Station in Dalian, China (121°7′48″ E, 38°49′12″ N). LY-3 was chosen as the control group to compare with YN-9 for cold tolerance assessment. All leaves of LY-3 perished after cold acclimation and freezing treatment, but only 5–30% YN-9 showed symptoms of yellowing and withering (Figure 3a). The DGCI value of LY-3 dropped from 70% to 45% after cold treatment, while the DGCI value of YN-9 increased from 67% to 80%, which was 1.78 times that of LY-3 (Figure 3b). All these results implied that YN-9 was significantly more cold-tolerant than LY-3. 

### 2.3. Read Trimming and Mapping

A total of 18 cDNA libraries were synthesized from RNA samples of two zoysiagrass biotypes (YN-9 and LY-3) collected at different treatment times (before treatment, after cold acclimation, and after freezing treatment) with three biological replicates, and then sequenced using the HiSeq X-ten. A total of 841,324,594 high-quality paired-end clean reads with read length of 150 bp, Q20 (the proportion of nucleotides with a quality score ≥ 20 in all nucleotides) of 95.03%, and GC content of 51.58% were yielded after filtering and trimming. Using Hisat2, the clean reads were mapped to the *Z. japonica* reference genome with an average mapping rate of 87.14% (Table S2).

### 2.4. DEGs after Cold Treatment

**Figure 4:**
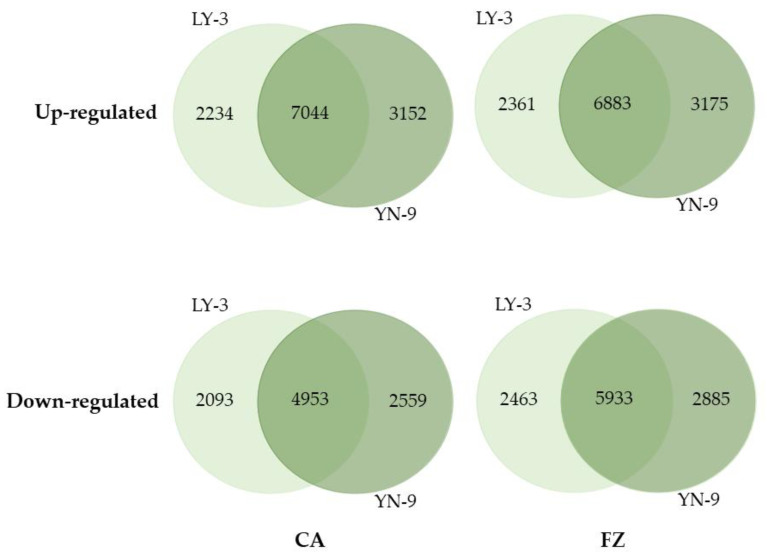
Venn diagram of up-regulated (log_2_FC >1 and *p*-value adjusted < 0.05) and down-regulated genes (log_2_FC < −1 and *p*-value adjusted < 0.05) of LY-3 and YN-9 after cold acclimation (CA) and freezing (FZ) treatment.

After cold acclimation (at 4 °C for 3 days), 9278 and 10196 genes were up-regulated in LY-3 and YN-9 leaves, respectively; 7046 and 7512 genes were down-regulated in LY-3 and YN-9 leaves, respectively. Among these genes, 7044 up-regulated and 4953 down-regulated genes were shared by the two Zoysia biotypes, whereas 3152 up-regulated and 2559 down-regulated genes were unique to YN-9 (Figure 4). After freezing treatment (at −4 °C for 1 day), 9244 and 10058 genes were up-regulated in LY-3 and YN-9 leaves, respectively; 8818 and 8396 genes were down-regulated in LY-3 and YN-9 leaves, respectively. Among these genes, 6883 up-regulated and 5933 down-regulated genes were shared by the two Zoysia biotypes, whereas 3175 up-regulated and 2885 down-regulated genes were unique to YN-9 (Figure 4).

**Figure 5:**
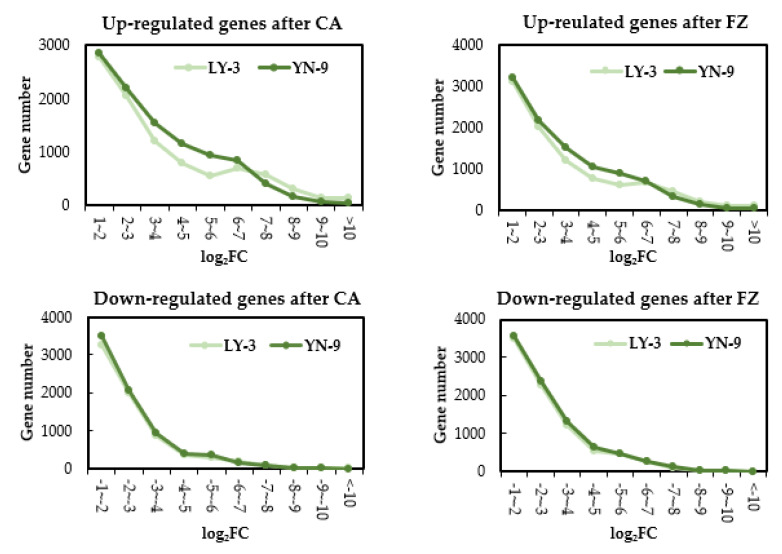
Distribution of up-regulated (log_2_FC > 1 and *p*-value adjusted < 0.05) and down-regulated genes (log_2_FC < −1 and *p*-value adjusted < 0.05) of LY-3 and YN-9 after cold acclimation (CA) and freezing (FZ) treatment in different log_2_FC intervals.

YN-9 had more differentially expressed genes than LY-3 following cold acclimation or freezing treatment, particularly those that were up-regulated (Figure 4). The distribution of up-regulated genes in different log_2_Fold Change (log_2_FC) intervals revealed that when log_2_FC was between 3 and 6, the difference in the number of up-regulated genes between the two biotypes expanded (Figure 5). As a result, DEGs for downstream GO and KEGG enrichment analyses were selected based on multiple log_2_FC thresholds instead of one single log_2_FC threshold.

### 2.5. Genes Enriched in Pigmentation GOs 

#### 2.5.1. HD-Zop IV

**Figure 6:**
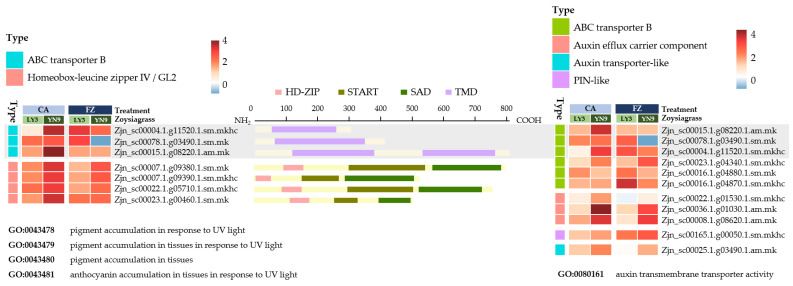
Expression variations and sequence structure of genes involved in pigment accumulation and auxin transmembrane transport in LY-3 and YN-9 after cold acclimation and freezing treatment. The heatmaps were drawn based on log_2_FC values. The grey rectangle highlighted the genes shared by GOs of pigment accumulation and auxin transmembrane transport. The protein domains were predicted with Pfam database using Interproscan.

When the screening criteria for up-regulated genes were elevated to log_2_FC > 2 and *p*-value adjusted < 0.05, genes that were uniquely up-regulated in YN-9- following cold acclimation were enriched in four Biological Process GO categories associated with pigment accumulation, which were GO:0043478, GO:0043479, GO:0043480 and GO:0043481. These entries all comprise seven genes, three of which belong to the ABC Transporter B (ABCB) family and four of which belong to the Homeobox-leucine Zipper IV (HD-ZIP IV) subfamily (Figure 6). 

**Figure 7:**
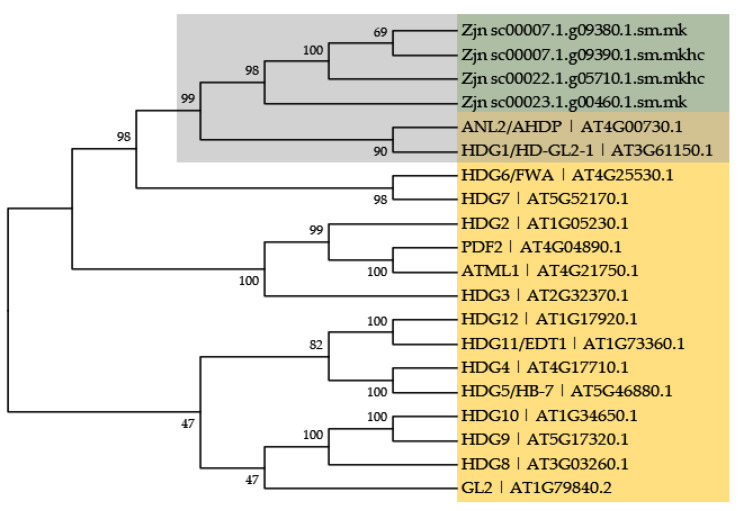
Phylogenetic analysis of the four *Zoysia japonica* HD-ZIP IV proteins (in green) associated with pigmentation and 16 *Arabidopsis thaliana* HD-ZIP IV members (in yellow). Multiple sequence alignment was performed using MUSCLE with default settings; Phylogenetic tree was constructed using Neighbor-Joining method with 1000 bootstrap replicates.

The four pigmentation-related *HD-ZIP IV* genes encode protein sequences with typical HD-ZIP IV structural features, HD-ZIP-START-SAD (Figure 6), and these protein sequences showed very high similarity with *Arabidopsis* ANL2 (AT4G00730.1) and HDG1 (AT3G61150.1) according to their BLAST results against TAIR protein database and multiple sequence alignment with *Arabidopsis* HD-ZIP IV members (Figure S2 and Table S3). Phylogenetic analysis of these four proteins with 16 *Arabidopsis* HD-ZIP IV members also clustered them together with ANL2 and HDG1, supported by a bootstrap value of 99% (Figure 7).

#### 2.5.2. ABCB Transmembrane Transporter

According to a BLAST search through the RAP and Swissprot database, the three ABCB genes from the pigmentation GOs were homologous to ABCB 1 or 19 (Table S4). However, only one or two transmembrane domains (TMDs) were found in the three genes, indicating a lack of complete gene structure as reported in ABCB members, which typically contain two TMDs and two nucleotide-binding domains (NBDs) [38,39,40,41]. These three genes were also associated with two auxin transport-related GOs, “auxin transmembrane transporter activity” and “auxin efflux transmembrane transporter activity”, which were also enriched with genes uniquely up-regulated in YN-9 (log_2_FC > 1 and *p*-value adjusted 0.05) (Figure 6). An *ABCB8* homolog “Zjn_sc00023.1.g04340.1.sm.mkhc” was also found to be persistently up-regulated following cold acclimation and freezing treatment. 

### 2.6. Expression Variations of Anthocyanin-Synthesis Genes

Using BLAST against the rice anthocyanin-synthesis (ATH-syn) gene sequences, 45 matched genes were retrieved from the zoysiagrass database. Seven of these were found to be false positive genes after being cross-checked against the TAIR, RAP, and Swissprot databases. Among the remaining 38 ATH-syn genes, 3 encode Anthocyanidin synthase (ANS), 5 encode Chalcone synthase (CHS), 2 encode Caffeic acid 3-O-methyltransferase (COMT), 3 encode Dihydroflavanol-4-reductase (DFR), 6 encode Flavanone 3-hydroxylase (F3H), 6 encode Phenylalanine ammonia-lyase (PAL), and 13 encode UDP-glucosyltransferase (UGT) (Table S5).

**Figure 8:**
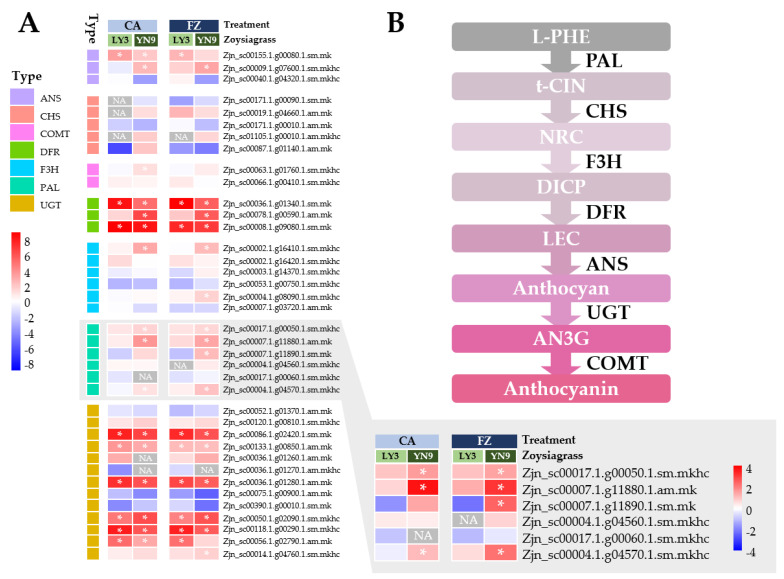
Expression variations of anthocyanin-biosynthesis genes in LY-3 and YN-9 after cold acclimation (CA) and freezing (FZ) treatment. (**A**) Heatmap based on log_2_FC of anthocyanin-biosynthesis genes, * indicates up-regulated genes with log_2_FC > 1 and *p*-value adjusted < 0.05. (**B**) Global picture of anthocyanin biosynthesis in plant cells.

LY-3 had 9 ATH-syn genes that were significantly up-regulated (log_2_FC > 1 and *p*-value adjusted < 0.05) following cold acclimation and freezing treatments, whereas YN-9 had 16 ATH-syn genes that were significantly up-regulated following cold acclimation and freezing treatments, respectively. The differences in gene expression changes between the two zoysiagrass biotypes were most evident in *PAL* genes, with three and four of the total six *PALs* in YN-9 significantly up-regulated after cold acclimation and freezing treatment, respectively, whereas no *PAL* genes in LY-3 showed significant up-regulation after either cold acclimation or freezing treatment (Figure 8).

### 2.7. Down-Regulation of Zinc Transporters in YN-9

**Figure 9:**
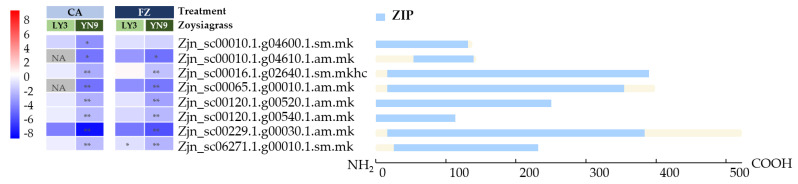
Expression variations and sequence structures of *ZIP* genes in LY-3 and YN-9 after cold acclimation and freezing treatment. The heatmaps were drawn based on log_2_FC values, * and ** indicate significant differences at *p*-values < 0.05 and 0.01, respectively. The sequence structures were drawn based on Interproscan analysis with PANTHER database.

Genes uniquely down-regulated in YN-9 (log_2_FC < −2 and *p*-value adjusted < 0.05) after cold acclimation were enriched in three GOs associated with zinc ion transport (GO0005385: zinc ion transmembrane transporter activity, GO0071577: zinc ion transmembrane transport, GO0006829: zinc ion transport), which all includes the same eight genes. According to a BLAST search in the Swissprot database, these genes encode proteins that are homologous to zinc transporters 3, 7, or 8, or Fe^2+^ transport protein 1 (Table S6). The Interproscan results also verified that all the eight genes contained the gene structure of zinc-regulated transporters and iron-regulated transporter-like proteins (ZIPs) (Figure 9). According to DEGSeq2 results, the expression levels of the eight *ZIP* genes in YN-9 decreased by 5-354 times after cold acclimation and by 3–70 times after freezing treatment. Seven of these genes remained significantly down-regulated in YN-9 after freezing treatment and were included in GO0005385 (zinc ion transmembrane transporter activity) which was among the GOs enriched with genes uniquely down-regulated in YN-9 after freezing treatment, while only one of them was significantly down-regulated in LY-3 after freezing treatment (Figure 9). 

## 3. Discussion

### 3.1. Anthocyanin Accumulation and Cold-Tolerance in YN-9

Anthocyanins are a group of flavonoid-type secondary metabolites found throughout the plant kingdom. They play a crucial role in plant reproduction by attracting pollinators and seed dispersers through pigmentation in flowers, fruits and vegetative tissues [42], and they also serve as protectants against a variety of abiotic and biotic stresses [23,43,44,45,46,47]. Anthocyanins accumulate in response to abiotic stresses such as drought, cold, and high light intensity. Low temperature, in particular, enhances anthocyanin accumulation, the abundance of anthocyanin-biosynthesis enzymes, and the expression of anthocyanin-biosynthesis genes in several plant species [21,23,24,25,43,48,49,50,51,52,53,54]. Although the exact mechanisms by which anthocyanins enhance cold tolerance is undetermined, a positive association between anthocyanin accumulation and cold tolerance has been well established in a variety of plant species [22,23,24,55,56]. Here, a zoysiagrass biotype YueNong No. 9 (YN-9) was collected in Lvshun West Station in Dalian, China (121°7′48″ E, 38°49′12″ N), where the average annual winter temperature ranges from −3 °C to −4 °C and the lowest temperature can reach −18 °C. As a result, we hypothesize that YN-9 is a cold-tolerant biotype that evolved as a result of long-term natural low-temperature screening. In comparison to LanYin No. 3 (LY-3), the most extensively utilized *Z. japonica* cultivar for urban greening and sports fields in southern China, YN-9 showed a larger portion of red to reddish–purple leaves, higher foliage anthocyanin content, and enhanced chilling tolerance when exposed to the natural low temperature through the winter in Guangzhou (Figure 2). Given the positive correlation between anthocyanin content and cold tolerance, we speculate that the ability to accumulate anthocyanins more efficiently at low temperatures is a cold-acclimation mechanism evolved by YN-9 to cope with the severe winter weather in its growing site, allowing it to acquire better cold tolerance than LY-3. The extent to which anthocyanin accumulation contributes to YN-9’s cold tolerance and the molecular mechanism by which anthocyanin influences low-temperature responses in YN-9 remain unanswered questions that will be a major focus of our future research.

### 3.2. Enhanced Expressions of Genes Associated with Anthocyanin Accumulation in YN-9

#### 3.2.1. Anthocyanin-Biosynthesis Genes

The primary regulators of anthocyanin biosynthesis are structural and regulatory genes [57,58,59,60,61,62,63,64,65,66,67,68,69,70,71]. The structural genes encode enzymes involved in anthocyanin biosynthesis, including phenylalanine ammonia lyase (PAL), cinnamate 4-hydroxylase (C4H), chalcone synthase (CHS), flavanone 3-hydroxylase (F3H), dihydroflavonol 4-reductase (DFR), anthocyanidin synthase (ANS), and UDP-glucose: flavonoid 3-O-glucosyltransferase (UFGT) [42]. Here, up-regulation of anthocyanin-biosynthesis genes were detected in both LY-3 and YN-9 after cold acclimation and/or freezing treatment. YN-9 had a larger amount of up-regulated genes than LY-3, and these genes that were uniquely up-regulated in YN-9 mainly belonged to *PALs* (Figure 8). PAL is a rate-limited enzyme engaged in the phenylalanine metabolism and catalyzes the conversion of L-phenylalanine to trans-cinnamic acid in the anthocyanin biosynthesis pathway [42]. There is enough evidence to suggest that the expression levels of *PAL* genes are associated to anthocyanin accumulation in plant tissues [72,73,74,75,76,77].The *pal1 pal2* mutant *Arabidopsis* was reported to accumulate little anthocyanin pigments when plants were grown in under-fertilized and relatively low-temperature conditions, suggesting a redundant and important role of these two genes in anthocyanin biosynthesis [78]. More particularly, *PAL* genes have been shown to play a specialized role in anthocyanin biosynthesis in response to low temperature, through investigation of these genes using gene expression profiling, enzymatic activity assessment, omic survey and promoter functional analysis. A previous research has shown that low temperature can trigger transcription of *PAL* genes, which leads to enhanced PAL activity in *Arabidopsis* [79]. An integrated transcriptomic and proteomic analysis indicated that two anthocyanin-biosynthesis genes *EjPAL* and *EjANS* might play a vital role in the cold response of *Eriobotrya japonica*. The *PAL* promoter in *Fagopyrum tataricum* (P*FtPAL*) was shown to be capable of conferring cold-induced expression through the investigation of the GUS activity changes in P*FtPAL*::GUS transformed tobacco leaves at low temperature [80]. Here, we found that *PAL* genes were uniquely up-regulated in YN-9 after cold acclimation and freezing treatment, while no *PAL* gene was observed to be up-regulated in LY-3 either after cold acclimation or freezing treatment. The increased low-temperature sensitivity of *PAL* gene may be one of the factors boosting anthocyanin production in YN-9, resulting in a higher anthocyanin content and better cold-tolerance than LY-3. 

#### 3.2.2. HD-IV Transcription Factors

Plant HD-ZIP transcription factors share a structural character of a leucine zipper motif down-stream of the homeodomain (HD) and can be classified into four subfamilies: HD-ZIP I, HD-ZIP II, HD-ZIP III, and HD-ZIP IV [81]. HD-ZIP IV subfamily was also known as HD-ZIP GL2 or simply GL2 family after its first identified member, the *Arabidopsis* GLABRA2 protein (AT1G79840). HD-ZIP IV members consist of a START (steroidogenic acute regulatory protein-related lipid transfer) domain connected to a conserved SAD (START-adjacent domain) and is primarily engaged in root growth, plant cell differentiation, trichome formation, and anthocyanin accumulation [82,83,84,85]. Here, genes uniquely up-regulated in YN-9 (log_2_FC > 2 and *p*-value adjusted < 0.05) were enriched in four GO categories associated with pigment accumulation (Figure 6). Four of these genes, Zjn_sc00007.1.g09380.1.sm.mk, Zjn_sc00007.1.g09390.1.sm.mkhc, Zjn_sc00022.1.g05710.1.sm.mkhc and Zjn_sc00023.1.g00460.1.sm.mk belong to HD IV (HD–GLABRA2 group) and all shared high resemblance with TAIR database is AtANL2 (AT4G00730) (Figure 7), which has been reported to be strongly associated with anthocyanin accumulation and transport in *Arabidopsis*. *Arabidopsis anl2* mutants accumulate significantly lower levels of anthocyanin in subepidermal and epidermal cells in the shoot and have numerous additional cells between the epidermal and cortical layers in the root [86,87]. It is possible that the *ANL2* homologs in zoysiagrass shared similar function and promoted anthocyanin accumulation in leaves of YN-9.

### 3.3. Enhanced Expressions of Anthocyanin Transport Genes in YN-9

Anthocyanins are synthesized in cytosol and endoplasmic reticulum membrane system, then transported into vacuoles via ABC transporters located in tonoplast [39,88,89,90,91,92]. In this study, three genes that were uniquely up-regulated in YN-9 lay in the overlap of auxin transport and pigmentation accumulation GOs. Although the expressions of these genes increased dramatically in YN-9 after cold acclimation, their expressions dropped back or even showed down-regulation after freezing treatment. In LY-3, although the expressions of these genes were less up-regulated than in YN-9 after cold acclimation, they continued to rise after freezing treatment. This implies that these genes in both YN-9 and LY-3 may respond to low temperatures, but at distinct rates and patterns. These genes were found to be the most similar to ABCB1 or 19 when compared to databases. ABCB1 and 19 are auxin efflux carriers involved in the accumulation of anthocyanin [93,94]. However, Interproscan analysis indicated that the proteins produced by these three genes do not have the complete ABCB structural domain. As a result, it was unclear if they truly play a role in anthocyanin transport and accumulation in zoysiagrass.

According to a study on Zijuan Tea (*Camellia sinensis* var. *kitamura*), the abundance of the ABC transporter B8 (ABCB8) as well as its mRNA expression level in purple leaves was significantly higher than in green leaves, implying that ABCB8 may be involved in the active transport of anthocyanin from cytoplasm to vacuoles, resulting in anthocyanin accumulation in cell vacuoles [95]. The homologous gene of this *ABCB8* in zoysiagrass, “Zjn_sc00023.1.g04340.1.sm.mkhc”, was also uniquely over-expressed in YN-9 both after cold acclimation and freezing treatment, implying a possible role of this gene in low-temperature-induced anthocyanin transport and accumulation in cell vacuoles (Figure 6). 

### 3.4. Down-Regulation of Zinc Transporters Genes in YN-9

Zinc is essential in the formation of chlorophyll, auxin and some carbohydrates, conversion of starches to sugars and its presence in plant tissue helps the plant to withstand cold temperatures [96]. According to a study on anthocyanin-rich purple corn, interacting with Zn/alginate slowed chemical degradation of anthocyanin [97]. GO Enrichment analysis showed that genes uniquely down-regulated in YN-9 were enriched in GO categories associated with zinc ion transport. The expression levels of the eight genes included in these GOs were not only significantly down-regulated after cold acclimation, but remained so after freezing treatment (Figure 9). Previous studies showed that genes in rice that were homologous to those eight genes were engaged in the distribution of Zn^2+^ from tissue to tissue [98,99]. Activation and over-expression of *OsZIP8* altered the zinc distribution in rice plants, causing lower levels in shoots and mature seeds, but an increase in the roots. When applied with excess zinc, transgenic rice contained less zinc in their shoots but accumulated more in the roots [98]. As a result, we postulated that down-regulation of *ZIPs* in YN-9 prevented Zinc efflux from leaf cells to other tissues, and the zinc ions maintained in the leaf cells interacted with anthocyanin and protected them from degradation.

### 3.5. A Proposed Regulation Network of Anthocyanin Accumulation in YN-9

**Figure 10:**
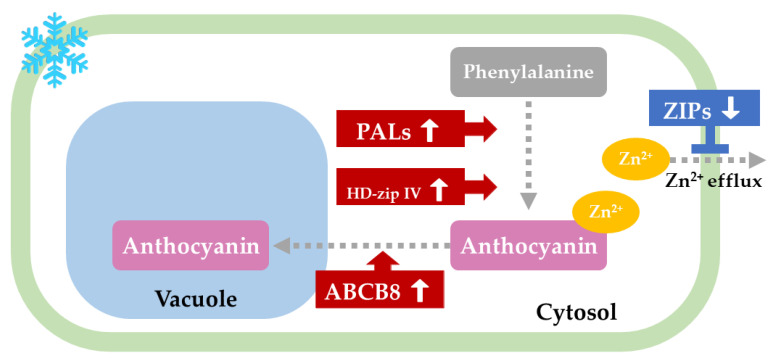
A proposed schematic representation of how low temperature-induced fluctuations of transcript abundance regulate anthocyanin synthesis, transport, and accumulation in YN-9 leaf cells. **→** and **┤** indicate positive and negative regulation of biological processes, respectively.

Different levels of tissue pigmentation were observed across zoysiagrass cultivars, for example, ‘Zenith’, ‘Millock’, and ‘Greenzoa’ have green spikes and stolons, while ‘Anyang-jungji’, ‘Meyer’, and ‘Senock’ develop purple colors in the same tissues, and anthocyanin accumulation is most likely to be responsible for this purple pigmentation [10,100,101,102]. Although a positive correlation has been well established between anthocyanin accumulation and cold tolerance in many plant species [22,23,24,55,56], no research has yet correlated anthocyanin accumulation with cold tolerance in zoysiagrass. Here, we discovered that YN-9 outperformed LY-3 in terms of cold endurance (Figure 3), and the leaves exhibited purple pigmentation and anthocyanin accumulation under natural low temperature (Figure 2). According to transcriptomic profiling, YN-9 showed a series of gene responses favoring anthocyanin accumulation after both cold domestication and freezing treatments, including the up-regulation of *PALs* favoring anthocyanin synthesis, the up-regulation of *HD-ZIP IV* genes favoring anthocyanin accumulation, the up-regulation of *ABCB8* favoring anthocyanin translocation from cytosol to vacuole, and the down-regulation of *ZIPs* which slowed the efflux of zinc ions from leaf cells, and therefore improved the chemical stability of anthocyanin within the cells (Figure 10). All of these findings are consistent with YN-9’s capacity to accumulate anthocyanin more effectively during the winter, suggesting that low temperature-induced gene responses that improve anthocyanin accumulation may be a coping strategy that YN-9 adopted to endure the harsh winter in northeast China (Figure 1). 

### 3.6. Future Outlook

Much more research is required to validate the proposed network, for example, a more detailed profiling of the expression fluctuations of candidate genes in zoysiagrass over time after being exposed to low temperature using Real-Time PCR, a more comprehensive investigation on the correlation between cold tolerance and anthocyanin accumulation in larger populations of zoysiagrass, a survey of trait segregation in the YN-9 progenies, functional analysis to verify the impact of the candidate genes on anthocyanin synthesis and cold tolerance by altering their expressions in zoysiagrass, such as gene over-expression in LY-3 using gene transformation, or gene knock-out/knockdown in YN-9 using CRISPR-Cas9/RNA interference. Given that genetic engineers have attempted to improve anthocyanin accumulation and cold tolerance in several plant species by introducing anthocyanin biosynthesis genes or regulatory transcription factors (TFs) [103,104,105], this work provides not just novel plant material for zoysiagrass breeding, but also new insights and potential genes for engineering breeding. 

## 4. Materials and Methods

### 4.1. Plant Materials

The *Zoysia japonica* biotype YueNong No. 9 (YN-9) were collected from Lvshun west station, Dalian, China (38°49′12″ N, 121°7′48″ E) on 7 July 2019 (Figure 1). The *Z. japonica* cultivar LanYin No. 3 (LY-3) were provided by Guangzhou Landscaping Company. Both zoysiagrasses were propagated vegetatively with stolons in a soil mixture of 1/2 Jiffy soil (Jiffy Products International AS, Stange, Norway) and 1/2 vermiculite in the College of Agriculture, South China Agricultural University (23°9′38″ N, 113°21’32″ E). The zoysiagrass biotypes were exposed to the natural low temperature in Guangzhou from October, 2020 to February 2021. The cold acclimation and freezing treatment of the zoysiagrass biotypes were performed in September 2021.

### 4.2. Anthocyanin Assay

The assessment of anthocyanin content in zoysiagrass leaves was developed from the method reported by Oancea et al. in 2012 [50]. Here, 0.5 g fresh leaves were grounded with liquid nitrogen and homogenized using extraction buffer of ethanol: 0.1 mol/L HCl (95:15). Extraction was facilitated by occasional shaking for 2 h. The obtained extracts were filtered and centrifuged at 8000 rpm at 4 °C for 10 min. The absorbance of the solution was measured using a spectrophotometer at 530 nm, 620 nm and 650 nm. The anthocyanin content was calculated using the following equation:

$$C=\frac{A\times V}{a\times l\times w}$$

where: C: anthocyanin content (mg/100g fresh weight)A: (OD_530 nm_ − OD_620 nm_) − 0.1(OD_650 nm_ − OD_620 nm_); V: volume of tested solution; a: molar absorption coefficient; l: path-length (cm); w: sample fresh weight (100 g).

### 4.3. Cold tolerance Assessment 

LY-3 and YN-9 sods of 7.5 cm in diameter were transplanted into pots of 10 cm × 10 cm × 14 cm, and filled with a soil mixture of 1/2 Jiffy soil and 1/2 vermiculite. One month after transplanted to the pots, both zoysiagrass biotypes were selected for cold treatment. The zoysiagrass was first subjected to a 3-day cold acclimation at 4 °C, followed by a 2-day freezing treatment at −4 °C. The temperature was progressively raised to 25 °C at a rate of 2 °C per hour, and the grasses were grown at this temperature for 2 additional weeks to assess the survival rate and dark green color index (DGCI) values. The entire treatment was carried out at 60% humidity under a 16/8 h (light/dark) photoperiod with a photon flux density (PFD) of 180 μmol·m^−2^·s^−1^.

The DGCI value is an indicator of dark green color that is calculated from the average of the converted HSB values: the greater the value, the more the leaf color is associated with dark green [106,107]. Each pot was photographed before and after treatment at the same time and in the same location with the same lighting. ImageJ (https://imagej.nih.gov/ij/, accessed on 29 December 2021) was used to identify RGB values in each photo, which were then converted into hue (H), saturation (S), and brightness (B). The DGCI values of each image were calculated using the following equation:

$$\mathrm{DGCI}=\frac{\left[\frac{H\;-60}{60}+\left(1-\;S\right)+\left(1-\;B\right)\right]}{3}$$

### 4.4. RNA Extraction, cDNA Library Construction and Illumina Sequencing

Fresh leaves were harvested for RNA extraction three times: once before treatment, once when cold acclimation was complete, and once after a 24 h freezing treatment. Total RNA was isolated using RNAprep Pure Plant Kit (TianGen Biotech, Beijing, China) from the collected leaves of LY-3 and YN-9, respectively. The RNA was electrophoresed on 1% agarose gels for monitoring RNA degradation and contamination. RNA purity, concentration, and integrity were further assessed using Qubit2.0 (Thermo Scientific, Waltham, MA, USA) and Agilent 2100 (Agilent Technologies, Santa Clara, CA, USA). A total of 18 RNA-Seq libraries (2 zoysiagrasses × 3 sampling times × 3 independent biological replicates) were constructed using the NEBNext^®^ Ultra™ Directional RNA Library Prep Kit for Illumina^®^ (NEB, USA), following the manufacturer’s instructions. RNA-seq libraries were sequenced using the Illumina HiSeq X-ten platform at Beijing Genomics Institute.

### 4.5. RNA Seq Analysis

Raw sequencing reads were processed with a perl script to remove adapters and primers, and discard reads containing over 5% ambiguous base N or over 50% low quality bases (quality score < 10). The clean reads were mapped to the reference genome of *Zoysia japonica* cv. Nagirizaki downloaded from *Zoysia* Genome Database (http://zoysia.kazusa.or.jp/, accessed on 29 December 2021) using HISAT2 [108,109]. Read counts were quantified using StringTie v2.0.4 [110]. Pairwise gene expression comparisons were performed using DESeq2 v1.26.0 [111]. Genes with FDR < 0.05 and absolute value of log_2_ fold change (log_2_FC) > 1 were identified as differentially expressed genes (DEGs). DEGs were then selected based on multiple log_2_FC thresholds for GO and KEGG enrichment using the clusterProfiler R package [112]. The clean reads were deposited to Sequence Read Archive (SRA) database at National Center for Biotechnology Information (NCBI) under BioProject PRJNA788220. 

### 4.6. Anthocyanin-Synthesis Gene Profiling

The rice anthocyanin-synthesis gene locus IDs were collected from an article published in 2021 by Ling et al. [113], and the gene sequences were obtained from the Rice Annotation Project Databases (RAP-DB, https://rapdb.dna.affrc.go.jp/tools/dump, accessed on 29 December 2021). BLAST was used to extract matching sequences from the zoysiagrass database, which were then compared with the TAIR, RAP-DB, and Swissprot databases to exclude false-positive sequences. 

### 4.7. Phylogenetic Analysis

To determine the homology between the 4 *Z. japonica* HD-ZIP IV proteins and AtHD-GL2 as well as AtANL2, 16 HD-ZIP IV protein sequences of *Arabidopsis thaliana* were aligned with the 4 *Z. japonica* proteins using MUSCLE with default settings. Phylogenetic tree was constructed from the multiple alignments using Neighbor-Joining method with 1000 bootstrap replicates.

### 4.8. Statistical Analysis

All morphological and physiological data were analyzed using the IBM SPSS Statistics 23.0 software. Significance of differences between samples or treatments were evaluated by Duncan’s multiple range test.

## 5. Conclusions

YueNong-9 (YN-9) is a *Zoysia japonica* biotype collected from northeast China where the winter temperature can drop to −18 °C. Using morphological, physiological, and transcriptomic approaches, the anthocyanin accumulation and cold tolerance were determined in YN-9 and LanYin-3 (LY-3), a popular *Z. japonica* cultivar in southern China. YN-9 demonstrated greater cold tolerance and anthocyanin accumulation under low temperature compared to LY-3. RNA-seq analysis revealed unique gene expression variations enhancing anthocyanin biosynthesis, transport, and accumulation, including up-regulation of *PALs*, *HD-ZIP IVs*, and *ABCB8*, and down-regulation of *ZIPs*. As a result, YN-9 is an ideal candidate for breeding cold-tolerant *Z. japonica* cultivars, and its unique low-temperature-induced anthocyanin accumulation and gene responses provide ideas and candidate genes for the study of low-temperature tolerance mechanisms and genetic engineering breeding. In the future, more comprehensive gene expression profiling and molecular approaches such as gene over-expression and knock out/knock down will be necessary to validate the putative roles of the aforementioned candidate genes in anthocyanin accumulation and cold tolerance in zoysiagrass.

## Figures and Tables

**Table 1:** Comparison of leaf blade length and width between YN-9 and LY-3.

Genotype	2nd Leaf		3rd Leaf	
	Length (cm)	Width (mm)	Length (cm)	Width (mm)
**LY-3**	10.19 ± 1.78	4.02 ± 0.55	11.93 ± 2.59	4.05 ± 0.58
**YN-9**	12.47 ± 2.28 **	3.92 ± 0.41	14.75 ± 2.59 **	3.93 ± 0.48

Note: ** indicates a significant difference (*p*-value < 0.01) between LY-3 and YN-9.

## Data Availability

The clean reads produced by transcriptomic sequencing of YueNong No. 9 and LanYin No. 3 were deposited to Sequence Read Archive (SRA) database at National Center for Biotechnology Information (NCBI) under BioProject PRJNA788220.

## Supplementary Materials

The following are available online at https://www.mdpi.com/article/10.3390/plants11030429/s1, Figure S1: *Zoysia japonica* ecotype Yuenong No. 9 (YN-9) collected from the edge of the tall fescue (*Festuca arundinacea*) lawn at Lvshun West Station, Dalian, China, Figure S2: Multiple sequences alignment of the four *Zoysia japonica* HD-Zip IV proteins associated with pigmentation and 16 *Arabidopsis thaliana* HD-Zip IV members, Table S1: Winter Temperature and Weather in Tianhe District, Guangzhou City, 2020–2021, Table S2: Clean read statistics and mapping rates, Table S3: BLAST results of the four pigmentation related HD-Zip IV proteins in zoysiagrass against TAIR protein database, Table S4: BLAST results of differentially expressed genes associated with GO0043478 in zoysiagrass, Table S5: BLAST results of anthocyanin biosynthesis associated genes in zoysiagrass, Table S6: BLAST results of zinc transport genes in zoysiagrass.
