# Supplementary material for: A Survey of Enhanced Cold Tolerance and Low-Temperature-Induced Anthocyanin Accumulation in a Novel Zoysia japonica Biotype
Source: Plants (Basel). 2022 Feb 4;11(3):429. doi: 10.3390/plants11030429 (PMC8839389; doi:10.3390/plants11030429)
Supplement: Supplementary file 1 [file plants-11-00429-s001.zip › Figure_S1_YN-9_collection_site_info.pdf]

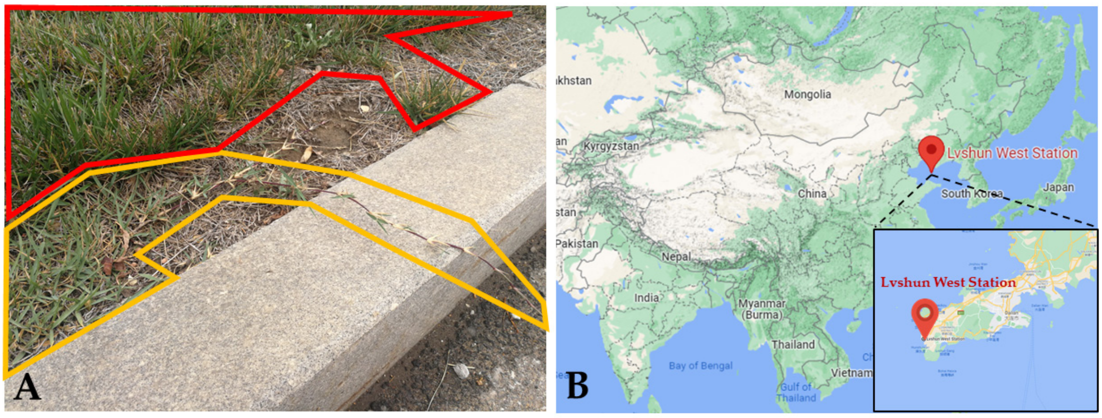

Figure S1 *Zoysia japonica* ecotype Yuenong No. 9 (YN-9) collected from the edge of the tall fescue (*Festuca arundinacea*) lawn at Lvshun West Station, Dalian, China. (A) YN-9 grown on the edge of the tall fescue lawn, the grasses with stolons surrounded by yellow lines are *Z. japonica*, the bushy grasses surrounded by red lines are tall fescue. (B) The location of Lvshun West Station in China.
