# Supplementary material for: A Survey of Enhanced Cold Tolerance and Low-Temperature-Induced Anthocyanin Accumulation in a Novel Zoysia japonica Biotype
Source: Plants (Basel). 2022 Feb 4;11(3):429. doi: 10.3390/plants11030429 (PMC8839389; doi:10.3390/plants11030429)
Supplement: Supplementary file 1 [file plants-11-00429-s001.zip › Figure_S2&Table_S3_HD-Zip_IV_genes.pdf]

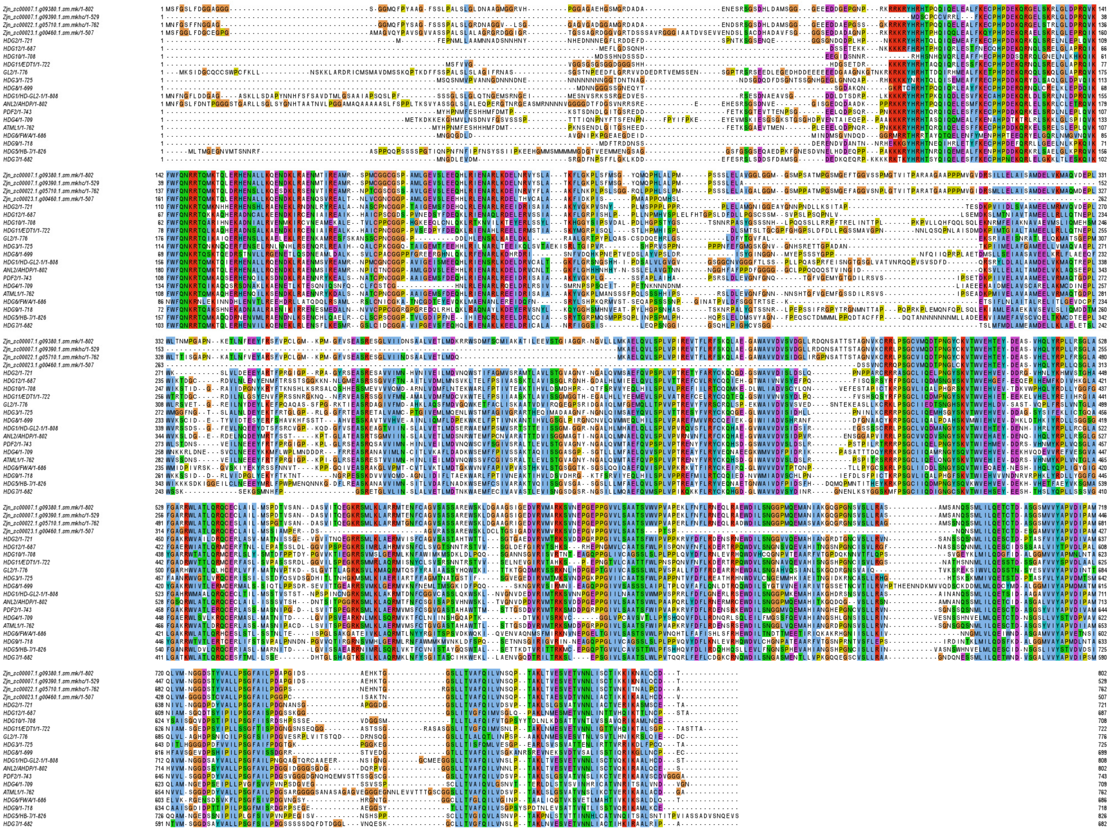

Figure S2 Multiple sequences alignment of the four *Zoysia japonica* HD-Zip IV proteins associated with pigmentation and 16 *Arabidopsis thaliana* HD-Zip IV members.

Table S3 BLAST results of the four pigmentation related HD-Zip IV proteins in zoysiagrass against TAIR protein database

query acc.ver	subject acc.ver	% identity	alignment length	mismatches	gap opens	q. start	q. end	s. start	s. end	evalue	bit score
Zjn_sc00007.1.g09380.1.sm.mk	AT3G61150.1	57.674	834	294	14	1	802	1	807	0	901
Zjn_sc00007.1.g09380.1.sm.mk	AT4G00730.1	57.026	854	263	18	1	802	1	802	0	895
Zjn_sc00007.1.g09390.1.sm.mkhc	AT3G61150.1	67.179	390	104	4	153	529	429	807	0	533
Zjn_sc00007.1.g09390.1.sm.mkhc	AT3G61150.1	79.31	116	24	0	11	126	128	243	1.93E-59	211
Zjn_sc00007.1.g09390.1.sm.mkhc	AT4G00730.1	68.407	383	103	5	153	529	432	802	3.61E-180	527
Zjn_sc00007.1.g09390.1.sm.mkhc	AT4G00730.1	83.478	115	19	0	11	125	152	266	2.37E-58	207
Zjn_sc00022.1.g05710.1.sm.mkhc	AT3G61150.1	54.426	836	278	17	1	762	1	807	0	817
Zjn_sc00022.1.g05710.1.sm.mkhc	AT4G00730.1	57.971	759	221	16	49	762	97	802	0	812
Zjn_sc00023.1.g00460.1.sm.mk	AT3G61150.1	50.893	336	70	6	264	506	473	806	3.35E-93	301
Zjn_sc00023.1.g00460.1.sm.mk	AT3G61150.1	50	260	101	7	1	248	1	243	9.86E-72	244
Zjn_sc00023.1.g00460.1.sm.mk	AT4G00730.1	51.713	321	70	6	269	506	483	801	8.45E-90	292
Zjn_sc00023.1.g00460.1.sm.mk	AT4G00730.1	50.179	279	92	5	1	246	1	265	9.00E-73	246

Note: AT3G61150.1 is the accession number of HDG1/HD-GL2-1 (HOMEODOMAIN-GLABRA2 1,homeodomain GLABROUS 1), AT4G00730.1 is the accession number of ANL2/AHDP (ANTHOCYANINLESS 2, ARABIDOPSIS THALIANA HOMEODOMAIN PROTEIN)
