# Supplementary material for: A Survey of Enhanced Cold Tolerance and Low-Temperature-Induced Anthocyanin Accumulation in a Novel Zoysia japonica Biotype
Source: Plants (Basel). 2022 Feb 4;11(3):429. doi: 10.3390/plants11030429 (PMC8839389; doi:10.3390/plants11030429)
Supplement: Supplementary file 1 [file plants-11-00429-s001.zip › Table_S1_2020-2021winter_weather_record.pdf]

Table S1 Winter Temperature and Weather in Tianhe District, Guangzhou City, 2020-2021

Date	Temp. Max	Temp. Min	Weather
2020/10/2	33℃	23℃	Overcast~Cloudy
2020/10/3	33℃	24℃	Cloudy
2020/10/4	33℃	23℃	Overcast~Showers
2020/10/5	28℃	21℃	Cloudy
2020/10/6	25℃	21℃	Overcast~Cloudy
2020/10/7	25℃	21℃	Overcast~Cloudy
2020/10/8	24℃	21℃	Overcast~Cloudy
2020/10/9	26℃	21℃	Cloudy
2020/10/10	29℃	21℃	Cloudy
2020/10/11	31℃	21℃	Sunny~Cloudy
2020/10/12	31℃	22℃	Cloudy
2020/10/13	27℃	22℃	Overcast~Heavy rain
2020/10/14	27℃	23℃	Overcast
2020/10/15	30℃	23℃	Overcast~Cloudy
2020/10/16	30℃	21℃	Cloudy
2020/10/17	26℃	21℃	Cloudy
2020/10/18	27℃	20℃	Overcast~Cloudy
2020/10/19	26℃	19℃	Cloudy
2020/10/20	27℃	19℃	Sunny
2020/10/21	25℃	19℃	Cloudy
2020/10/22	26℃	19℃	Cloudy
2020/10/23	26℃	17℃	Cloudy
2020/10/24	26℃	18℃	Overcast~Cloudy
2020/10/25	28℃	20℃	Overcast~Cloudy
2020/10/26	31℃	19℃	Overcast~Sunny
2020/10/27	31℃	19℃	Cloudy
2020/10/28	27℃	19℃	Overcast~Showers
2020/10/29	22℃	19℃	Light rain~Cloudy
2020/10/30	24℃	20℃	Overcast~Cloudy
2020/10/31	27℃	19℃	Cloudy~Sunny
2020/11/1	29℃	20℃	Cloudy
2020/11/2	27℃	19℃	Overcast~Cloudy
2020/11/3	25℃	19℃	Cloudy~Sunny
2020/11/4	24℃	19℃	Sunny~Cloudy
2020/11/5	27℃	20℃	Sunny~Cloudy
2020/11/6	30℃	21℃	Sunny~Cloudy
2020/11/7	29℃	20℃	Sunny~Cloudy
2020/11/8	29℃	20℃	Cloudy~Sunny
2020/11/9	27℃	18℃	Cloudy~Sunny
2020/11/10	26℃	18℃	Cloudy~Sunny
2020/11/11	27℃	15℃	Sunny
2020/11/12	28℃	16℃	Sunny
2020/11/13	27℃	19℃	Sunny~Overcast
2020/11/14	22℃	21℃	Overcast
2020/11/15	23℃	19℃	Light rain~Cloudy
2020/11/16	30℃	20℃	Cloudy
2020/11/17	26℃	22℃	Cloudy
2020/11/18	30℃	23℃	Overcast~Cloudy
2020/11/19	31℃	23℃	Overcast~Cloudy
2020/11/20	30℃	22℃	Sunny~Light rain
2020/11/21	27℃	21℃	Overcast~Light rain
2020/11/22	30℃	20℃	Overcast~Cloudy
2020/11/23	23℃	18℃	Overcast~Cloudy
2020/11/24	26℃	19℃	Overcast~Sunny
2020/11/25	28℃	19℃	Sunny
2020/11/26	28℃	16℃	Cloudy~Sunny
2020/11/27	24℃	14℃	Sunny
2020/11/28	23℃	13℃	Cloudy
2020/11/29	20℃	14℃	Sunny~Cloudy
2020/11/30	21℃	13℃	Cloudy~Sunny
2020/12/1	22℃	14℃	Sunny
2020/12/2	22℃	13℃	Sunny~Cloudy
2020/12/3	19℃	12℃	Sunny
2020/12/4	17℃	10℃	Cloudy~Sunny
2020/12/5	19℃	11℃	Sunny
2020/12/6	21℃	12℃	Sunny
2020/12/7	22℃	11℃	Cloudy~Sunny
2020/12/8	22℃	12℃	Sunny~Cloudy
2020/12/9	21℃	14℃	Cloudy
2020/12/10	23℃	18℃	Overcast~Cloudy
2020/12/11	24℃	18℃	Cloudy
2020/12/12	23℃	18℃	Overcast~Cloudy
2020/12/13	22℃	12℃	Overcast~Light rain
2020/12/14	15℃	9℃	Light rain
2020/12/15	11℃	8℃	Overcast~Cloudy
2020/12/16	13℃	10℃	Overcast~Cloudy
2020/12/17	11℃	9℃	Overcast~Cloudy
2020/12/18	17℃	9℃	Cloudy
2020/12/19	14℃	9℃	Overcast~Cloudy
2020/12/20	16℃	10℃	Cloudy
2020/12/21	18℃	10℃	Cloudy

Date	Temp. Max	Temp. Min	Weather
2020/12/22	19℃	13℃	Cloudy~Light rain
2020/12/23	23℃	14℃	Overcast~Cloudy
2020/12/24	20℃	11℃	Cloudy
2020/12/25	16℃	15℃	Overcast~Cloudy
2020/12/26	22℃	16℃	Cloudy
2020/12/27	26℃	16℃	Cloudy~Sunny
2020/12/28	27℃	16℃	Sunny~Cloudy
2020/12/29	27℃	9℃	Cloudy
2020/12/30	14℃	5℃	Sunny
2020/12/31	15℃	3℃	Sunny
2021/1/1	17℃	7℃	Sunny
2021/1/2	18℃	10℃	Cloudy
2021/1/3	21℃	15℃	Cloudy
2021/1/4	23℃	11℃	Cloudy
2021/1/5	20℃	8℃	Cloudy
2021/1/6	13℃	5℃	Cloudy
2021/1/7	10℃	5℃	Cloudy
2021/1/8	13℃	8℃	Cloudy
2021/1/9	13℃	7℃	Cloudy
2021/1/10	11℃	6℃	Cloudy
2021/1/11	16℃	6℃	Cloudy~Sunny
2021/1/12	18℃	8℃	Sunny~Cloudy
2021/1/13	19℃	10℃	Light smog
2021/1/14	25℃	12℃	Light smog~Cloudy
2021/1/15	25℃	12℃	Cloudy
2021/1/16	18℃	7℃	Sunny
2021/1/17	18℃	6℃	Cloudy
2021/1/18	21℃	12℃	Cloudy~Light smog
2021/1/19	22℃	15℃	Cloudy~Light smog
2021/1/20	22℃	16℃	Light smog~Cloudy
2021/1/21	23℃	15℃	Light rain~Sunny
2021/1/22	25℃	14℃	Sunny
2021/1/23	23℃	14℃	Sunny~Cloudy
2021/1/24	26℃	14℃	Cloudy
2021/1/25	25℃	13℃	Sunny
2021/1/26	24℃	14℃	Cloudy
2021/1/27	20℃	11℃	Cloudy
2021/1/28	21℃	10℃	Sunny
2021/1/29	22℃	11℃	Sunny
2021/1/30	24℃	13℃	Sunny
2021/1/31	25℃	15℃	Cloudy~Sunny
2021/2/1	24℃	13℃	Sunny
2021/2/2	23℃	14℃	Sunny
2021/2/3	24℃	15℃	Sunny

Note: Temp. Max and Temp. Min indicates the highest and lowest daily temperature.
